# Context matters! The relationship between mother-reported family nutrition climate, general parenting, food parenting practices and children’s BMI

**DOI:** 10.1186/s12889-016-3683-8

**Published:** 2016-09-27

**Authors:** Sanne M. P. L. Gerards, Christina Niermann, Dorus W. M. Gevers, Nadine Eussen, Stef P. J. Kremers

**Affiliations:** 1 Department of Health Promotion, NUTRIM School for Nutrition and Translational Research in Metabolism, Maastricht University, Maastricht, The Netherlands; 2 Karlsruhe Institute of Technology, Karlsruhe, Germany

## Abstract

**Background:**

Efforts to explain children’s nutrition behavior or weight often involve investigating the parent-child relationship, typically studying the associations between food parenting practices (FPPs) and child outcomes. However, these behaviors are embedded in a broader system: general parenting (GP, the general emotional climate at home), and the family health climate (an aspect of the broader family system in the context of health). In the current study, we combined the parent-child measures of parenting (FPPs and GP) and the nutritional dimension of the family health climate (family nutrition climate, FNC) to get a broader view of how these concepts are interrelated. The current study had two aims: predicting FPPs using GP and FNC as predictor variables, and investigating the relationship between FPPs and children’s weight in different groups of parents, based on low and high GP and FNC scores.

**Methods:**

We collected cross-sectional data via an online survey panel. Mothers of 267 children aged 5–12 years filled out a questionnaire assessing demographics (e.g., children’s weight and height), GP, FPPs, and FNC. Bivariate correlation coefficients were calculated between all constructs. Structural equation modeling was performed to test the hypothesized relationships between GP, FNC and FPPs. Hereafter, different groups of parents were identified, using median split, based on a low or high score on GP or a low or high score on FNC. Bivariate correlation coefficients were calculated between FPPs and children’s BMI z-score for these different groups.

**Results:**

GP and FNC were consistently positively correlated (all r’s ≥.177), and both concepts were positively associated with healthy FPPs (all r’s ≥.214). In families with a positive context (i.e. scoring high on GP and on FNC), healthy FPPs were associated with lower BMI z-scores of the children (r -.229). This association was not found for children with a more negative family context.

**Conclusions:**

FNC and GP are valuable additional concepts to investigate relationships between FPPs and child outcomes. We recommend that more studies, next to investigating the parent-child system, include a measure of the broader family system, in order to get a broader view of the mechanisms explaining child health behaviors and weight status.

## Background

Children frequently eat unhealthy food. As a result, the amounts and types of food children consume are often not in line with current dietary recommendations [1–4]. For example, energy-dense diets, high consumption of sugar-sweetened beverages, large portion sizes and unhealthy eating patterns are risk factors for obesity [5]. Children’s nutrition behaviors are to a large extent influenced by their family and their parents [6]. Research in the field mostly focuses on two different parenting behaviors: general parenting (GP) and food parenting practices (FPPs). GP, also called parenting style, reflects the emotional climate (created by parental attitudes, beliefs and behaviors) in which parent-child interactions take place [7]. GP is often expressed as the extent to which parents provide structure (organize their child’s environment), nurturance (stimulate and recognize individuality) and behavioral control (supervise and manage their children’s activity) [8, 9]. Parents who score high on all three constructs are often described as authoritative parents. In contrast, FPPs are context-specific acts of parenting regarding children’s eating [7]. Examples of FPPs are parental rules regarding soft drink consumption or the availability of food at home.

Both parenting behaviors have been intensively investigated for their influence on children’s nutrition behaviors. Overall, authoritative parenting (or positive parenting) has been shown to be positively associated with children’s healthy eating behaviors, children’s physical activity levels, and lower BMI z-scores [10, 11]. It should be noted however that the impact of GP is rather small. GP is a distal variable and its effects are mediated by more proximal variables such as FPPs. As regards the effect of FPPs, some FPPs have been consistently associated with children’s healthy food intake (for example parental modeling and availability of healthy food) [12–14], while other FPPs were not consistently related to healthy food intake (for example highly controlling practices) [15]. Inconsistent findings might be explained by the fact that these studies did not assess the broader context (GP) in which these practices take place [16]. This is illustrated by the study by Sleddens and colleagues [16], who found that for children who were reared in a positive parenting context (in terms of nurturance and structure), encouragement and covert control were more effective in promoting healthy food intake than for children raised in a less positive context.

Both GP and FPPs are behaviors of parents aimed at influencing their child’s behaviors, making these behaviors aspects of the parent-child subsystem (that is, parent-child interaction). This subsystem is, however, embedded in a broader family context [17, 18], which consists of several subsystems, for example sibling and marital relationships and the family as a whole, with reciprocal influences between the different subsystems (individual, parent-child and family). Therefore, parenting behaviors and their effects on children’s behavior should be seen in the context of the family as a whole. This is also recognized in the Model of Family Reciprocal Determinism [19], which provides a framework for the influences of family environment on individual health behavior. By taking into account the family as a whole, different parameters become relevant.

One relevant concept is the family health climate (FHC) [20], reflecting an aspect of the broader family system in the context of health. The FHC is a family-level variable which can be defined as ‘the shared perceptions and cognitions concerning a healthy lifestyle within a family’ [20]. This variable is assumed to affect the health behavior of all family members and has been operationalized by a scale measuring the family physical activity climate and a scale measuring the family nutrition climate (FNC). The FNC was found to be associated with adolescents’ consumption of fruit, vegetables and salad [21].

Both the parent-child subsystem and the broader family system have been claimed to be important in influencing children’s energy balance-related behaviors [6, 22]. The aim of the current study was to examine the relationship between different aspects of the parent-child subsystem and the family system. We did this by examining the relationships between constructs reflecting different levels of the family environment: FPPs, GP and the FNC. In addition, we explored the relationship between FPPs and children’s BMI z-scores.

**Fig. 1:**
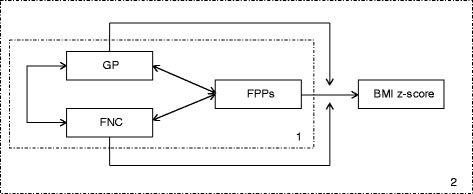
Assumed relations between family concepts. GP = general parenting, FPPs = food parenting practices, FNC = family nutrition climate; 1 = research question 1, 2 = research question 2

**Fig. 2:**
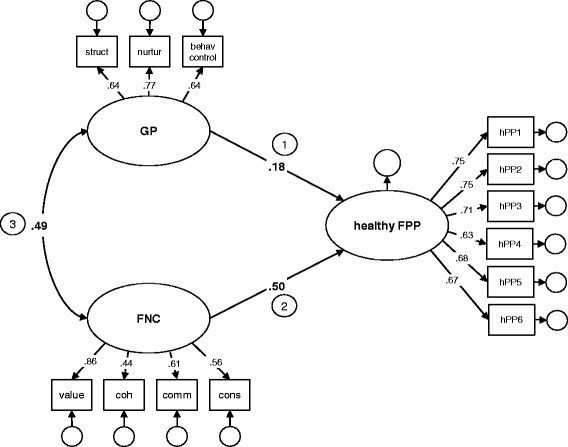
Model A. Relationship between general parenting (GP), family nutrition climate (FNC) and healthy food parenting practices (healthy FPPs)

The current study was intended to gain further knowledge regarding the influences of the family food environment on children’s nutrition behavior by exploring the underlying mechanisms. As prior research showed that FPPs and GP are relevant predictors of children’s weight status, we would like to extend this knowledge by investigating the added value of the broader family climate, operationalized by the FNC. In our theoretical model, the FNC adds to the relation between GP and healthy FPPs (Fig. 1). We assume that the pathways are bidirectional. However, for the current study, we considered FPPs as the outcome measure, since they are more proximal to the child’s behavior. The aims of the current study were twofold: (1) predicting FPPs using GP and FNC as predictor variables (see part 1 in Figs. 1) and (2) investigating the relationship between FPPs and BMI z-score in different groups of parents, based on GP scores (low and high) and FNC scores (low and high) (see part 2 in Fig. 1). Our hypothesis was that GP, FNC and FPPs are correlated, but different constructs. Furthermore, we expected that the relation between FPPs and BMI was different in different subgroups of parents (based on GP and FNC scores).

## Methods

### Study design and participants

The current study was based on an online survey with a cross-sectional design. Participants were invited to participate in the study via an online survey panel (Thesis tools, The Hague). This panel consists of participants who receive an invitation to participate in a survey once a month. The inclusion criterion was that participants had to have a least one child aged between 5 and 12 years. Participants were recruited until the desired sample size was reached. Participants did not receive any incentive for participation.

### Measures

#### Family health climate scale

The Family Health Climate Scale (FHC-scale) [20] consists of 31 items measuring the shared cognitions and perceptions of a healthy lifestyle within a family regarding nutrition (FNC) and physical activity (family physical activity climate). In the current study, we only included the FNC. The scale is tested for validity and reliability in prior research [20]. The scale can be filled out by all family members, but the in current study, the scale is filled out by mothers. The scale contains 17 items (α = .854) and consists of four subscales: cohesion, communication, value and consensus (see Table 1 for detailed information about the scales). A high score on the scale indicates a positive FNC. All items were introduced by ‘In our family…’. Answers were scored on a 4-point rating scale from totally disagree to totally agree.

**Table 1:** Overview of scales

Scales	N of items	Cronbach’s alpha	Example item
*FNC*	17	.854	
cohesion	5	.784	In our family we appreciate spending time together during meals
communication	5	.768	In our family we talk about which foods are healthful
value	4	.811	In our family it is normal to choose healthful foods
consensus	3	.847	In our family we rarely argue about food- or diet-related matters
*GP*	33	.818	
nurturance	12	.708	When my child is sad, I know what is going on with him/her
structure	12	.615	I help my child plan his/her activities for the day/week
behavioral control	9	.718	I correct my child when he/she breaks the rules
*FPPs*			
healthy FPPs	6	.854	I eat consciously healthy products in the presence of my child
covert FPPs	4	.671	I monitor what my child eats during the day
overt FPPs	2	.695	I have rules for my child about eating sweets and snacks
non-nutritive FPPs	2	.535	I give my child candy or snacks to ensure he/she feels better

*FPPs* food parenting practices, *FNC* family nutrition climate, *GP* general parenting

#### Translation procedure for FHC scale

Various experts at Maastricht University translated the FHC-scale into Dutch. We used an extensive translation procedure [23]. First, four bilingual experts (2 German native speakers and 2 Dutch native speakers) independently translated the questionnaire into Dutch. Inconsistencies were then discussed together until consensus was reached. This led to a first provisional version of the questionnaire. This version was translated back into German by a German native speaking expert who had not seen the questionnaire before. Again, consistencies and inconsistencies were discussed with the translators. Hereafter, the provisional version of the questionnaire was pretested among a target population of six parents, using cognitive interviewing (verbal probing techniques). As a result, some corrections were discussed with the translators to further adjust the questionnaire. The translated questionnaire was also submitted for approval to other experts at the Department of Health Promotion who are working in the field of nutrition and physical activity. Any remaining uncertainties were discussed with the developer of the FHC-scale (CN).

#### Comprehensive general parenting questionnaire

The Comprehensive General Parenting Questionnaire (CGPQ) was developed and validated in Dutch by Sleddens and colleagues [9]. The questionnaire consists of five GP constructs: nurturance, structure, behavioral control, coercive control and overprotection. In the current study, we included the constructs that have been shown to have a positive influence on child development: nurturance, structure and behavioral control (reflecting positive parenting) [9]. All items were scored on a 5-point Likert scale, ranging from ‘strongly disagree’ to ‘strongly agree’. See Table 1 for detailed information about the subscales.

#### Food parenting practices

FPPs were measured using the Dutch Comprehensive Snack Parenting Questionnaire (CSPQ) (Gevers DWM, Kremers SPJ, de Vries NK, van Assema P: Development of the Comprehensive Snack Parenting  Questionnaire: Test-retest reliability and discriminative value of constructs, submitted). The questionnaire was developed to measure 21 different FPPs related to snack intake: encouragement, rewarding, discussing, providing feedback, involving, educating, healthy modeling and avoidance of unhealthy modeling, availability of healthy foods, accessibility of healthy foods, visibility of healthy foods, limited availability of unhealthy foods, limited accessibility of unhealthy foods, structure and meal routines, permissiveness, rules, monitoring, instrumental feeding, emotional feeding and pressure to eat. Example items are: ‘I monitor what my child eats during the day’ (monitoring) and ‘I give my child candy or snacks to reward him/her when he/she has done something good’ (instrumental feeding). All these FPPs were measured using a single item with a 5-point Likert scale from ‘strongly disagree’ to ‘strongly agree’.

To extract factors of FPPs, exploratory factor analyses were performed using principal axis factoring with oblique Promax rotation [24, 25]. The requirements for exploratory factor analyses in this sample were fulfilled (Kaiser-Meyer-Olkin = .82, Bartlett’s test of sphericity *χ*^2^ (210) = 1630.19, *p* < .01). There were no correlations above .85 between any pair of items [26]. The Kaiser criterion (eigenvalue > 1) yielded five factors with eigenvalues greater than one. Using the initial factor solutions, items were removed step by step based on the following criteria: factor loading < .40, cross-loading > .30, communality < .30 and corrected item-scale correlation < .30 [26].

Based on these criteria, seven items were removed. Finally, four factors were extracted: healthy FPPs (6 items: availability of healthy foods, accessibility of healthy foods, encouragement, visibility of healthy foods, educating and meal routines), covert FPPs (4 items: accessibility of unhealthy foods, unhealthy modeling avoidance, healthy modeling and monitoring), overt FPPs (2 items: rules and structure) and non-nutritive FPPs (2 items: emotional and instrumental feeding) (see Table 1).

### Demographics

Participants were asked to report their gender, marital status, educational level, ethnicity, weight and height. Marital status was classified into (1) married/living together or (2) other. Educational level was categorized into (1) low (primary school, lower vocational education, lower secondary education, intermediate vocational education, higher general secondary education and university preparatory education) and (2) high (higher vocational education or university). Ethnicity was categorized into (1) Netherlands and (2) other. Weight and height were used to calculate body mass index (BMI). BMI scores were then categorized into: (1) underweight (BMI < 18.5), (2) normal weight (BMI 18.5–25.0), (3) overweight (BMI 25.0–30.0) and (4) obese (BMI >30.0).

Furthermore, participants were asked to report their children’s gender, age, weight and height. Children’s weight and height were used to calculate BMI (weight (kg)/height (m))^2^. Children’s BMI was recoded into BMI z-scores, compared to the 1997 national reference population (Fourth Dutch National Growth Study). Weight status was classified into healthy weight (5^th^–84^th^ percentile), overweight (85^th^–94^th^ percentile) and obesity (≥95^th^ percentile).

### Data analysis

Only participants who completed all items were included in the analyses. Furthermore, due to the small number of male participants (*N* = 8), men were excluded from the analyses. Means and frequencies of demographics were calculated using descriptive statistics. Pearson correlation tests were used to calculate correlations between GP, FNC, FPPs and BMI z-score of the child. Effects were interpreted using the classification defined by Cohen: small effect (*r* = 0.1–0.3), medium effect (*r* = 0.3–0.5), or large effect (*r* ≥ 0.5). Hereafter, multivariate regression models were conducted to determine the effect of GP and FNC on FPPs. These models were corrected for the following covariates: marital status, educational level, BMI, and ethnicity mother, and gender, age and BMI z-score of the child. These analyses were conducted using IBM SPSS Statistics version 21 (IBM Corp., NY, USA).

Structural equation modeling was performed with IBM AMOS 22 (IBM Corp., NY, USA) using maximum likelihood estimation to test the hypothesized relationships between GP (nurturance, structure and behavioral control as indicators), the FPP factors and FNC. The fit indices χ^2^/df, CFI, SRMR and RMSEA were used to assess the goodness of fit, a good fit being indicated by 0 ≤ χ^2^/df ≤ 2, .97 ≤ CFI ≤ 1, 0 ≤ SRMR ≤ .05 and RMSEA ≤ .05, and an acceptable fit by 2 < χ^2^/df ≤ 3, .95 ≤ CFI < .97, .05 < SRMR ≤ .10 and .05 < RMSEA ≤ .08 [27]. The bootstrapping procedure was used to obtain bias-corrected 95 % confidence intervals. Standardized values were used to interpret the results.

In order to answer our 2^nd^ research question, four different groups of parents were identified, using median split, based on a low or high score on GP (= sum score of nurturance, structure and behavioral control; low < 4,3, high > 4,3; group 1 and 2) or a low or high score on FNC (low < 3, high > 3; group 3 and 4). Hereafter, four additional different groups of parents were identified based on the combination of low or high scores on GP and low or high scores on FNC (group a-d). Bivariate Pearson correlation coefficients were then calculated between FPPs and children’s BMI z-score for these eight different groups.

## Results

### Participants

In total, 267 mothers of children aged 5–12 years filled out the questionnaires. Demographic characteristics of both parents and children are shown in Table 2. The study population consisted of mothers who were mainly married and were mostly Dutch. About half of the children were male and the children were on average 8.8 years old.

**Table 2:** Characteristics of the study population

	Number	Percent	Mean	SD
*Characteristics of mothers*				
Marital status				
Married/living together	244	91.4		
Other	23	8.6		
Educational level				
Low	71	26.6		
High	196	73.4		
BMI			24.63	4.31
Underweight	2	0.8		
Normal weight	162	61.1		
Overweight	78	29.4		
Obese	23	8.7		
Ethnicity				
Netherlands	252	94.4		
Other	15	5.6		
*Characteristics of children*				
Gender				
Male	136	50.9		
Female	131	49.1		
Age			8.78	2.19
BMI z-score			−0.35	1.27
Underweight	33	12.6		
Normal weight	192	73.6		
Overweight	36	13.8		

### Correlations between GP, FNC and FPPs

Bivariate correlation coefficients between the GP subscales (i.e. nurturance, structure and behavioral control), FNC subscales (i.e. value, cohesion, communication and consensus), FPP factors (i.e. healthy FPPs, covert FPPs, overt FPPs and non-nutritive FPPs) and child BMI z-score are shown in Table 3. All indicators of GP were positively related to all subscales of FNC, with small effect sizes. Moreover, structure and nurturance were positively correlated to healthy FPPs (small to medium effects) and overt FPPs (small effect), and negatively to non-nutritive FPPs (small effect). Behavioral control was positively related to healthy FPPs (small effect sizes), covert FPPs and overt FPPs. Overall, FNC subscales were positively correlated (small to medium effect sizes) to healthy FPPs, covert FPPs and overt FPPs, and were negatively correlated to non-nutritive FPPs. None of the GP and FPPs factors were correlated to BMI z-scores of the child, while the FNC subscales for communication and consensus were significantly negatively correlated to children’s BMI z-scores (small effects).

**Table 3:** Correlation coefficients between GP, FNC subscales, FPPs and BMI z-score

		M (SD)	Pearson correlation coefficients										
			2	3	4	5	6	7	8	9	10	11	12
1	GP structure	4.00 (.40)	**.493**	**.409**	**.274**	**.218**	**.180**	**.319**	**.214**	.058	**.236**	**−.214**	.016
2	GP nurturance	4.49 (.32)	-	**.480**	**.246**	**.257**	**.177**	**.296**	**.320**	.032	**.262**	**−.123**	.007
3	GP behavioral control	4.25 (.40)	-	-	**.215**	**.179**	**.205**	**.241**	**.214**	**.124**	**.239**	.048	−.014
4	FNC value	3.13 (.47)	-	-	-	**.383**	**.543**	**.476**	**.437**	**.332**	**.198**	**−.250**	−.115
5	FNC cohesion	3.52 (.41)	-	-	-	-	**.138**	**.282**	**.290**	.085	**.187**	**−.146**	−.020
6	FNC communication	2.75 (.51)	-	-	-	-	-	**.287**	**.324**	**.428**	**.176**	−.069	**−.131**
7	FNC consensus	2.76 (.64)	-	-	-	-	-	-	**.252**	**.141**	.051	**−.205**	**−.131**
8	Healthy FPPs	4.49 (.51)	-	-	-	-	-	-	-	**.222**	**.276**	**−.260**	−.095
9	Covert FPPs	3.12 (.83)	-	-	-	-	-	-	-	-	**.270**	.073	−.059
10	Overt FPPs	4.13 (.81)	-	-	-	-	-	-	-	-	-	−.057	−.079
11	Non-nutritive FPPs	1.96 (.78)	-	-	-	-	-	-	-	-	-	-	.096
12	BMI z-score child	−.35 (1.27)	-	-	-	-	-	-	-	-	-	-	-

*GP* general parenting, *FNC* family nutrition climate, *FPPs* food parenting practices; bold numbers are statistically significant (*P* < .05)

### Model of GP, FNC and FPPs

GP and FNC were included in a model to predict FPPs (four factors: healthy FPPs, covert FPPs, overt FPPs and non-nutritive FPPs), corrected for relevant covariates (Table 4).

**Table 4:** Standardized regression coefficients on the prediction of FPPs using GP and FNC

	Healthy FPPs	Covert FPPs	Overt FPPs	Non-nutritive FPPs
Model 1:				
GP	**.187**	−.007	**.293**	−.083
FNC	**.327**	**.232**	.111	−.102

All analyses were corrected for marital status, educational level, BMI, ethnicity mothers and for gender, age and BMI-z children. Bold numbers were statistically significant

Hereafter, four structural equation models were analyzed reflecting our conceptual model (see part 1 in Fig. 1) of GP, FNC and the four FPP factors. The model with healthy FPPs as outcome variable is displayed in Fig. 2 (Model A). The fit indices of the four models and the standardized path coefficients are displayed in Table 5.

**Table 5:** Standardized regression coefficients and fit indices of the models

	Path				Fit indizes				
	1	2	3						
	β (95 % CI)	β (95 % CI)	r (95 % CI)	R^2^	*χ* ^2^, df, p	*χ* ^2^/df	SRMR	CFI	RMSEA, (95 % CI), *p*
Model A Healthy FPPs	**.183 (.022–.353)**	**.498 (.323–.684)**	**.490 (.316–.632)**	.37	98.923, 60, .001	1.649	.045	.967	.049, (.031, .066), .506
Model B Covert FPPs	−.190 (−.415–.028)	**.686 (.486–.898)**	**.507 (.350–.640)**	.37	125.913, 41, .000	3.071	.075	.878	.088, (.071, .106), .000
Model C Overt FPPs	**.389 (.199–.601)**	.130 (−.079–.308)	**.489 (.289–.639)**	.22	46.957, 24, .004	1.915	.052	.960	.059, (.032, .084), .269
Model D Non-nutritive FPPs	.010 (−.218–.261)	**−.321 (−.583 – −.113)**	**.488 (.292–.639)**	.10	56.471, 24, .000	2.353	.060	.934	.071, (.047, .096), .071

*FPPs* food parenting practices; Bold numbers are statistically significant (*P* < .05)

Both of the distal constructs, GP and FNC, were consistently positively correlated (Fig. 2, path 3). However, the strength of the relationship between the constructs differed, depending on the FPP factor. GP and FNC were both positively related to healthy FPPs (Model A). Compared to GP, FNC was more strongly related to healthy FPPs. The overall model fit was acceptable and the model explained 37 % of the variance in healthy FPPs. As regards covert FPPs (Model B), FNC had a significant effect while the effect of GP was not significant. However, this model did not have a satisfactory fit. Overt FPPs (Model C) was dependent on GP but not on FNC. FNC was negatively related to non-nutritive FPPs (Model D), while GP was unrelated to non-nutritive FPPs. Both models C and D had an acceptable overall model fit, and explained 22 and 10 % of the variance in FPPs, respectively.

### Relationship between food parenting practices and children’s BMI z-score in different contexts

FPP-BMI z-score relationships appear to be strongest in the context of groups 4 (high GP), a (low FNC, high GP) and c (high FNC, high GP; Table 6). In the groups scoring either low or high on FNC and the groups scoring low or high on GP (Groups 1–4), we found no statistically significant correlation between FPPs and children’s BMI z-score child. However, we found that for children who were raised in a positive system (high scores on GP and high scores on FNC, group c), healthy FPPs were associated with lower child BMI z-score. This association was not found for children who were raised in either a positive parenting context or in a healthy nutrition climate. The correlations between healthy FPPs and children’s BMI z-score in the other subgroups were not significant. Also, none of the other FPP factors were statistically significantly correlated to children’s BMI z-scores.

**Table 6:** Pearson correlation coefficients between FPPs and children’s BMI z-score for different groups of parents, based on FNC (low and high) and GP (low and high)

	N	Healthy FPPs	Covert FPPs	Overt FPPs	Non-nutritive FPPs
Whole sample	261	−.095	−.095	−.079	.096
Group 1	132	−.042	−.088	−.090	.090
Low FNC					
Group 2	129	−.075	.017	−.031	.077
High FNC					
Group 3	132	−.079	−.029	−.012	.064
Low GP					
Group 4	129	−.124	−.087	−.171	.134
High GP					
Group a	51	−.039	−.189	−.222	.171
Low FNC, High GP					
Group b	81	−.035	−.030	−.015	.032
Low FNC, Low GP					
Group c	78	**−.229**	−.034	−.144	.111
High FNC, High GP					
Group d	51	.057	.074	.069	.073
High FNC, Low GP					

*FPPs* food parenting practices, *FNC* family nutrition climate, *GP* general parenting; bold numbers are statistically significant (*P* < .05)

## Discussion

### Main findings

Traditionally, observational research has mainly focused on the parent-child interaction when trying to explain children’s nutrition behaviors. For example, the effects of FPPs and GP have been intensively investigated. However, as the parent-child subsystem is only one part of the family, the broader family context should be taken into account, to get a more complete picture of the working mechanisms of the broader family system [17, 18]. The current study investigated the relationships between the parent-child subsystem (GP and FPPs) and the broader family system, as operationalized by the concept of FNC. FNC is a relatively new concept measuring the family climate regarding nutrition. This measure is developed as a family level variable affecting the health behavior of family members. This variable is different from traditional measures, which often measure only one part of the system: parent-child interactions. Only two prior studies investigated this concept. The first study was a validation study of the FHC instrument [20]. In the second study, FHC showed to be associated with adolescents’ physical activity behavior and nutrition behavior and they found that this was mediated by adolescents’ intrinsic motivation [21]. The current study was the first to investigate the link between FHC (in this case FNC) and parental measures.

### Relationship between different family-related constructs

With regard to the bivariate correlations, we found modest positive correlations between indicators of GP and healthy FPPs, and negative correlations with unhealthy FPPs. These findings were similar to those of other studies (e.g., Sleddens et al. [16]). However, the correlations were different in our full models, in which the correlations between GP and FPPs were corrected for the FNC. We found that healthy FPPs were more strongly related to FNC (compared to GP) whereas overt control FPPs were more strongly related to GP (compared to FNC). This could be due to the fact that healthy FPPs, for example meal routines, can be considered to reflect parents’ attitudes and values around eating, which is an element of the FNC. In contrast, overt control FPPs (rules and structure) correspond to behaviors that constitute GP. In the full model, non-nutritive FPPs (instrumental and emotional feeding) were negatively correlated to FNC. This is in line with what we expected, since these practices are detrimental to children [16, 28]. Moreover, we found no association between GP and non-nutritive FPPs (in the corrected model). However, in the bivariate correlations, small correlations were found between structure and nurturance and non-nutritive FPPs. This is somewhat similar to what Philips et al. [29] found: a small negative correlation between emotional eating and structure and behavioral control. Moreover, Sleddens et al. [16] found in their study that nurturance and structure were associated with lower use of instrumental and emotional feeding. Covert control FPPs were bivariately correlated to three of the four FNC subscales, but this correlation did not appear in the full model.

### Relationship between family-related constructs and children’s BMI z-score

Neither the GP constructs, nor the FPPs were correlated to BMI z-score. Other studies also found no or small relations between GP and children’s weight see for example [10, 15]. This can be explained by the fact that GP is a distal variable, which is relatively far away from children’s weight in the causal chain [9]. In the total sample, the associations between FPPs and child BMI z-scores were in the expected direction (i.e. healthy FPP were negatively related to BMI z-scores and non-nutritive FPPs were positively related to BMI z-score). The relations were however not statistically significant. Other energy balance-related behaviors in children may weaken the direct association between food parenting and children’s weight [30]. However, the association between healthy FPPs and children’s weight was statistically significant even in the relatively small subsample of children living in a family with an optimal climate. This finding is rather promising, since it provides us with provisional evidence that FPPs can have an impact on children’s weight as long as the context is optimal. These results underline the importance of incorporating the child’s family context in interventions that aim at sustained effects on children’s weight.

### Strengths and limitations

This is the first study in which measures of the parent-child subsystem were combined with a variable at the family level, which we assume to be necessary to explain the mechanism underlying the influence parents have on their children’s nutrition behavior. We recommend that more studies combine these measures in order to get a comprehensive understanding of factors which impact on children’s nutrition behaviors and weight. Another strength of our study is that we used validated instruments to measure GP, FPPs and the FNC.

It should be noted, however, that we chose to include a relatively new questionnaire on FPPs, developed and validated by Gevers and colleagues (unpublished). The added value of this questionnaire is that it measures the full spectrum of FPPs related to snack intake, which is not done by most other questionnaires measuring FPPs, such as the Child Feeding Questionnaire [31] and the Parental Feeding Style Questionnaire [32]. We chose to not use the items as isolated types of FPPs, but extracted four latent factors of the 21 items. However, these factors were data-driven and need to be validated in future studies.

A limitation of the current study was that we did not measure children’s energy balance-related behaviors and children’s health condition and that BMI was self-reported by the mothers. We recommend that future studies investigating the association between parenting measures and the family context include measures of children’s behaviors and ideally, BMI should be measured objectively. Also, we did not ask for the economic status of the family (although we know the educational level of the mother) and whether the participants lived in urban or rural areas.

Another limitation of this study is that multiple testing can lead to incorrect observations of significant results. It is therefore also important to interpret patterns in the data, without explicitly focusing on the significant correlations.

We measured solely the maternal view of GP, FPPs and the FHC, and this might differ from the children’s perspective [33, 34]. Nor did we include paternal views on these concepts, which may differ from maternal views [35]. Due to the small number of fathers (*N* = 8), we decided to exclude these from our analyses.

It should be noted that the group size of the groups of the sub group analyses is rather small and that these results should be interpreted with caution.

Finally, this was a cross-sectional study, which limits the possibility to investigate causal relationships. It is very desirable that future studies address this topic using longitudinal data.

### Recommendations

Regarding observational research, we advocate an approach in which research combines the traditional parenting concepts with the broader family context. It would be very interesting to relate both measures to children’s health behaviors. Although we think that both measures are important in explaining children’s health behaviors, studies measuring general parenting and not the health context, may lack relevant information. Ideally, the family health climate is measured by assessing this within all family members.

As regards interventional research, we think that intervention designers should not only focus on changing FPPs, but take into account that the context of these practices matters substantially. Reaching optimal effects requires intervening on the family system.

## Conclusion

The family nutrition climate is a valuable addition to general parenting and food parenting practices. Based on the current findings, we expect that these three types of environmental influences all are of added value in explaining children’s lifestyle behaviors and children’s weight. System-based thinking is relatively new in the field of family based research. We recommend that more studies, next to investigating the parent-child system, include a measure of the broader family system, in order to get a broader view of the mechanisms explaining child health behaviors and weight status.

## Abbreviations

CGPQ: Comprehensive general parenting questionnaire
CSPQ: Comprehensive snack parenting questionnaire
FHC: Family health climate
FNC: Family nutrition climate
FPPs: Food parenting practices
GP: General parenting

## References

[1] Nielsen SJ, Rossen LM, Harris DM, Odgen CL. Fruit and vegetable consumption of U.S. Youth, 2009-2010. NCHS Data Brief 2014(156):1-8. 25027507

[2] Walther J, Aldrian U, Stuger HP, Kiefer I, Ekmekcioglu C (2014). Nutrition, lifestyle factors, and mental health in adolescents and young adults living in Austria. Int J Adolesc Med Health.

[3] Keast DR, Fulgoni VL, Nicklas TA, O’Neil CE (2013). Food sources of energy and nutrients among children in the United States: National Health and Nutrition Examination Survey 2003–2006. Nutrients.

[4] Gevers DW, Kremers SP, de Vries NK, van Assema P. Intake of energy-dense snack foods and drinks among Dutch children aged 7–12 years: how many, how much, when, where and which? Public Health Nutr 2015:19(1): 83-92. 10.1017/S1368980015000877 PMC10270797 25850560

[5] Rennie KL, Johnson L, Jebb SA (2005). Behavioural determinants of obesity. Best Pract Res Clin Endocrinol Metab.

[6] Gerards S, Kremers S (2015). The role of food parenting skills and the home food environment in children’s weight gain and obesity. Curr Obes Rep.

[7] Darling N, Steinberg L (1993). Parenting style as context: an integrative model. Psychol Bull.

[8] Skinner E, Johnson S, Snyder T (2005). Six dimensions of parenting: a motivational model. Parenting.

[9] Sleddens EF, O’Connor TM, Watson KB, Hughes SO, Power TG, Thijs C, De Vries NK, Kremers SP (2014). Development of the comprehensive general parenting questionnaire for caregivers of 5-13 year olds. Int J Behav Nutr Phys Act.

[10] Pinquart M (2014). Associations of general parenting and parent-child relationship with pediatric obesity: a meta-analysis. J Pediatr Psychol.

[11] Sleddens EF, Gerards SM, Thijs C, De Vries NK, Kremers SP (2011). General parenting, childhood overweight and obesity-inducing behaviors: a review. Int J Pediatr Obes.

[12] Pearson N, Biddle SJ, Gorely T (2009). Family correlates of fruit and vegetable consumption in children and adolescents: a systematic review. Public Health Nutr.

[13] van der Horst K, Oenema A, Ferreira I, Wendel-Vos W, Giskes K, van Lenthe F, Brug J (2007). A systematic review of environmental correlates of obesity-related dietary behaviors in youth. Health Educ Res.

[14] Rasmussen M, Krolner R, Klepp KI, Lytle L, Brug J, Bere E, Due P (2006). Determinants of fruit and vegetable consumption among children and adolescents: a review of the literature. Part I: Quantitative studies. Int J Behav Nutr Phys Act.

[15] Wardle J, Carnell S (2007). Parental feeding practices and children’s weight. Acta Paediatr.

[16] Sleddens EF, Kremers SP, Stafleu A, Dagnelie PC, De Vries NK, Thijs C (2014). Food parenting practices and child dietary behavior. Prospective relations and the moderating role of general parenting. Appetite.

[17] Cox MJ, Paley B (2003). Understanding families as systems. Curr Dir Psychol Sci.

[18] Cox MJ, Paley B (1997). Families as systems. Annu Rev Psychol.

[19] Taylor WC, Baranowski T, Sallis JF (1994). Family determinants of childhood physical activity: a social-cognitive model.

[20] Niermann C, Krapf F, Renner B, Reiner M, Woll A (2014). Family health climate scale (FHC-scale): development and validation. Int J Behav Nutr Phys Act.

[21] Niermann CY, Kremers SP, Renner B, Woll A (2015). Family health climate and adolescents’ physical activity and healthy eating: a cross-sectional study with mother-father-adolescent triads. PLoS One.

[22] Kremers S, Sleddens E, Gerards S, Gubbels J, Rodenburg G, Gevers D, van Assema P (2013). General and food-specific parenting: measures and interplay. Child Obes.

[23] Beaton DE, Bombardier C, Guillemin F, Ferraz MB (2000). Guidelines for the process of cross-cultural adaptation of self-report measures. Spine.

[24] Russell DW (2002). In search of underlying dimensions: the use (and abuse) of factor analysis in personality and social psychology bulletin. Personal Soc Psychol Bull.

[25] Reise SP, Waller NG, Comrey AL (2000). Factor analysis and scale revision. Psychol Assess.

[26] Worthington RL, Whittaker TA (2006). Scale development research: a content analysis and recommendations for best practices. Couns Psychol.

[27] Schermelleh-Engel K, Moosbrugger H, Müller H (2003). Evaluating the fit of structural equation models: tests of significance and descriptive goodness-of-fit measures. Methods Psychol Res Online.

[28] Vereecken CA, Keukelier E, Maes L (2004). Influence of mother’s educational level on food parenting practices and food habits of young children. Appetite.

[29] Philips N, Sioen I, Michels N, Sleddens E, De Henauw S (2014). The influence of parenting style on health related behavior of children: findings from the ChiBS study. Int J Behav Nutr Phys Act.

[30] Larson N, Story M (2013). A review of snacking patterns among children and adolescents: what are the implications of snacking for weight status?. Child Obes.

[31] Birch LL, Fisher JO, Grimm Thomas K, Markey CN, Sawyer R, Johnson SL (2001). Confirmatory factor analysis of the child feeding questionnaire: a measure of parental attitudes, beliefs and practices about child feeding and obesity proneness. Appetite.

[32] Wardle J, Guthrie CA, Sanderson S, Rapoport L (2001). Development of the children’s eating behaviour questionnaire. J Child Psychol Psychiatry.

[33] Rebholz CE, Chinapaw MJ, van Stralen MM, Bere E, Bringolf B, De Bourdeaudhuij I, Jan N, Kovacs E, Maes L, Manios Y (2014). Agreement between parent and child report on parental practices regarding dietary, physical activity and sedentary behaviours: the ENERGY cross-sectional survey. BMC Public Health.

[34] van Assema P, Glanz K, Martens M, Brug J (2007). Differences between parents’ and adolescents’ perceptions of family food rules and availability. J Nutr Educ Behav.

[35] Berge JM, MacLehose RF, Meyer C, Didericksen K, Loth KA, Neumark-Sztainer D (2015). He said, she said: examining parental concordance on home environment factors and adolescent health behaviors and weight status. J Acad Nutr Diet.

